# Genomic epidemiology of *Mycobacterium tuberculosis* in Wales

**DOI:** 10.1038/s41598-025-15076-8

**Published:** 2025-08-24

**Authors:** Nicole Pacchiarini, Felicity Simkin, Mark Postans, George Ahern, Jiao Song, Clare Brown, Josie Smith, Catie Williams, Matthijs Backx, Daniel Thomas, Thomas R. Connor, Christopher Williams

**Affiliations:** 1 Communicable Disease Surveillance Centre (CDSC), Public Health Wales, Cardiff, Wales UK; 2 Public Health Genomics, Public Health Wales, Cardiff, Wales UK; 3 Cardiff University School of Biosciences, Cardiff University, Cardiff , Wales UK; 4 Pathogen Genomics Unit, Public Health Wales, Cardiff, Wales UK; 5 Public Health Wales Microbiology Cardiff, University Hospital of Wales, Heath Park Cardiff, Cardiff, Wales UK

**Keywords:** Tuberculosis, Whole genome sequencing, Genomic cluster, United Kingdom, Wales, Tuberculosis, DNA sequencing, Epidemiology

## Abstract

Identification of factors contributing to tuberculosis (TB) transmission can guide targeted measures to reduce morbidity. Varying findings for factors associated with TB genomic clustering exist. We describe *Mycobacterium tuberculosis* strain diversity, drug-resistance, and ongoing transmission in Wales using single nucleotide polymorphisms (SNP)-based typing to infer lineage and clusters. TB cohort data on isolates from Welsh residents from 2012 to 2022, patient level data from the National TB Surveillance System and SNP-based data, were merged. Descriptive epidemiology and logistic regression modelling were used to identify factors associated with genotypic clustering. 215 cases were included in the cluster analysis (66% male and 46% born outside of the UK); 115/215 belonged to 30 genomic clusters belonging to lineages 2–4. Most clusters corresponded to Lineage 4 and were distributed within South Wales. There were significant differences in the distribution of ethnicity, age group, and deprivation (Welsh Index of Multiple Deprivation, WIMD) in our sample compared to the Welsh population. Resistance to rifampicin and isoniazid and predicted resistance to ethambutol, aminoglycosides, pyrazinamide, and quinolone was low. Factors associated with increased odds of clustering included being UK-born and having pulmonary disease. Due to the identification of the above factors associated with TB genomic clustering, as well as the differences in ethnicity, age group, and WIMD quintile, prevention strategies for TB screening targeted towards these groups may be considered. Future work may evaluate the utility of additional control measures within these populations when the onset case in a genomic cluster has any of these characteristics.

## Introduction

The World Health Organisation (WHO) Global Tuberculosis Report 2024 estimates that globally, there are ten million new cases of Tuberculosis (TB) per year, despite TB being preventable and usually curable^1^. Further, the only single infectious agent to result in more fatalities is SARS-CoV-2^1^. In 2023, the United Kingdom (UK) had a TB incidence rate of 7.84 cases per 100,000 people (*n* = 5,298 notifications)^2^ with 4,716 new diagnoses and relapses in 2022 and a 7% case fatality ratio in 2022^1^. The number and rate of TB notifications in the UK in 2023 increased by 12.4% compared to 2022, the largest increase observed since 2000^2^. In 2023, Wales, a country with a low TB burden, reported a TB incidence rate of 2.7 cases per 100,000 people with this increasing to 6.9 cases within the most deprived regions, according to the Welsh Index of Multiple Deprivation (WIMD; see Methods for further details)^3^.

Those with health-related inequalities are at a disproportionate risk of TB^4^. TB risk factors include migration from areas of high-incidence, homelessness, alcoholism and illicit drug use, imprisonment, smoking and underlying illnesses such as diabetes, hepatitis B, hepatitis C, and human immunodeficiency virus (HIV)^1,5^. The population risk of acquiring TB may be influenced by the factors that contribute to TB transmission. Identifying these risk factors in Wales and taking targeted measures may help reduce TB morbidity. Varying findings for factors associated with genomic clustering exist within the literature, especially between low and high incidence countries^6^. Recent UK-wide studies have identified the following factors: TB lineage, being male, pulmonary disease, previous TB diagnosis, being UK-born, alcohol and drug misuse, imprisonment, being an asylum seeker, having mental health needs, high level of deprivation based on the Index of Multiple Deprivation (IMD; the official measure of relative deprivation for small areas in England, derived similarly to WIMD), homelessness, ethnicity, and younger age^7–10^. Other international studies have identified additional associations with: treatment failure, immigration status, and HIV infection (although some studies find no association between HIV and clustering risk)^6,11,12^.

In Wales, classical genotyping methods such as Spoligotyping and Mycobacterial Interspersed Repetitive Unit—Variable Number of Tandem Repeat (MIRU-VNTR) were used for epidemiological investigations of TB. In 2019, the Pathogen Genomics Unit (PenGU) at Public Health Wales (PHW) introduced Whole Genome Sequencing (WGS) for resistance calling and clustering. This allowed TB outbreaks to be determined with a higher resolution, including revealing genomic clusters proposed to reflect epidemiological links between patients and highlighting cross-border outbreaks^13,14^. TB has been classified into nine lineages with Lineages 4 (Euro-American) and 3 (Central Asian Strain) being the most common in the UK in 2010–2015^7^. As well as estimating strain diversity, WGS can be used to infer genotypic-based predictions of drug resistance. Drug-resistant TB is a public health crisis and health security threat, which complicates TB treatment and can result in poorer patient outcomes^15,16^. In the UK in 2023, 2.3% of all culture-confirmed TB notifications were MDR or rifampicin resistant; of which none were from Wales^2^. Some evidence exists that drug resistance, transmissibility, virulence, host response, disease site, severity and clinical outcome varies between TB lineages^17,18^.

We aim to provide an overview of the *Mycobacterium tuberculosis* strain diversity, drug-resistance and ongoing transmission in Wales using single nucleotide polymorphism (SNP)-based typing to infer TB lineage and cluster groups. We also summarised the geographical distribution of clusters, and factors associated with genomic clustering.

## Method

### Study design and case definition

Patients resident in Wales with a bacteriological confirmation of TB, diagnosed in Wales between 2012 and December 2022 who had started first line anti-TB treatment were retrospectively analysed.

### Ethics declaration

The data used within this study is enhanced surveillance utilising routinely collected data, in line with PHW statutory function and therefore ethics approval is not required. The use of named patient data in the investigation of communicable disease outbreaks and surveillance of notifiable disease is permitted under Public Health Wales’ Establishment Order. Data were held and processed under Public Health Wales’ information governance arrangements, in compliance with the Data Protection Act, Caldicott Principles and Public Health Wales guidance on the release of small numbers. No data identifying protected characteristics of an individual were released outside Public Health Wales.

### WGS cluster definition

WGS on culture confirmed samples of TB from residents in Wales is carried out by the PenGU. This commenced in 2019, set up involved retrospective sequencing of MIRU-VNTR clustered isolated from pre-2019; historical samples were obtained from storage and submitted for WGS. Information on WGS linked clusters of TB containing two or more Welsh cases is generated by the UK Health Security Agency (UKHSA) through the FindNeighbours (https://github.com/davidhwyllie/findNeighbour2), part of the Compass pipeline (https://github.com/oxfordmmm/CompassCompact; v1.7)^19^. There is a 12 SNP distance cut-off for clustering isolates in Wales. This threshold is the standard baseline across the UK and is defined in the UKHSA cluster investigation handbook, building on several key publications concluding that epidemiological links consistent with transmission did not exist between isolates with a > 12 SNPs difference, including Walker et al.^13,20,21^. Where the lineage for cases within the cluster analysis were unknown, the lineage of the other cases within the 12 SNP cluster was assigned^13^.

### WGS resistance calling

Resistance is currently defined into groups as per WHO guidelines: TB strains resistant to rifampicin and isoniazid (multidrug resistant (MDR)), strains that are MDR with added resistance to second line agents including fluoroquinolones (pre-extensively drug resistant (pre-XDR)), and strains fulfilling MDR definition with additional resistance to any fluoroquinolone and at least one Group A drug (extensively drug resistant (XDR))^22^. Information on resistance prediction is generated by an in-house interim pipeline within the PenGU, followed by a final result generated by the UKHSA through an in-house version of the Compass (COMplete Pathogen Analytical Software Solution) pipeline (https://github.com/oxfordmmm/CompassCompact; v1.7)^19^. As described previously, the latter leverages a custom mutation catalogue incorporating data informed by^23,24^, but remains to be updated to reflect the more comprehensive WHO catalogue of drug-resistance conferring mutations^25,26^. The data used in this study is taken from the Compass pipeline outputs^26^.

PHW reports resistance using WGS for rifampicin and isoniazid. Resistance prediction is also generated for ethambutol, aminoglycosides, pyrazinamide, and quinolone by the UKHSA pipeline but has not been validated for clinical use in Wales. WGS-based resistance status prediction is categorised as resistant, susceptible, unknown (the software for WGS-based genotypic testing could not determine the resistance level or the quality control criteria for the pipeline which calls resistance were not met) or not tested (WGS resistance testing was not performed). Where samples failed the quality control thresholds or were not tested using WGS-based genotypic testing, phenotypic testing results were used—this was undertaken by the Wales Centre for Mycobacteria. Resistance as reported by phenotypic testing is categorised as resistant, susceptible or not tested (phenotypic resistance testing was not performed).

### Exclusion criteria

Samples were excluded if they were from an individual not resident in Wales; were not notified to the National TB Surveillance System (NTBS); or were not *Mycobacterium tuberculosis*.

### Data sources

PHW links patient information from the NTBS, a surveillance system run by UKHSA that provides detailed information on the epidemiology of TB in England, Wales and Northern Ireland, with the sequence data from isolates. Clinical teams notify newly diagnosed cases and update information on treatment outcomes. All people diagnosed with TB in Wales are reported through NTBS. This linked data is merged with TB cohort data and patient level data from The UK Mycobacterial Surveillance Network System (MycobNet) and the WIMD. WIMD is the official measure of relative deprivation for small areas in Wales and is itself derived from eight indicators of deprivation (income, employment, health, education, access to services, housing, community safety and physical environment), with income and employment given the greatest weight^27^.

### Statistical analysis

The number of TB genomic clusters (< 12 SNPs) and their size range per characteristic (described below) was calculated. The geographical spread of clustered cases and drug resistance profiles for the sequenced/typed cases were investigated. Cases were eligible for the resistance analysis if either a WGS or phenotypic resistance result was available for at least one of the six drugs.

Demographic and other characteristics of the study population were as follows: age group (0–18, 19–24, 25–34, 35–44, 45–54, 55–64, 65+), sex (Male, Female), ethnic group (White, Bangladeshi, Indian, Pakistani, Asian – Other, Black, Chinese, Mixed/Other, unknown), country of birth (born in the UK, born outside of the UK, unknown), WIMD quintile of residency (1, 2, 3, 4, 5), site of disease (pulmonary, pulmonary and other sites, extra pulmonary), and the following variables, all of which had the levels yes, no, unknown: any social factor potentially associated with genomic clustering (imprisonment, homelessness status, drug misuse, alcohol misuse), HIV, hepatitis B, hepatitis C, diabetes, liver disease, kidney disease, immunosuppression, and previous infection. Fisher’s exact test was used to test for a univariate association between cases’ genomic clustering status (‘clustered’ versus ‘non-clustered’) and their distribution across each of the above characteristics. Fisher’s test was favoured over a Chi-squared independence test after model assumption checks revealed that for some comparisons, the expected values were below 5 in some contingency table cells.

The sample age, ethnicity, and WIMD quintile distributions were compared to the broader Welsh population using separate Chi-squared goodness-of-fit tests, with p-values computed via Monto Carlo simulation. Population distributions were obtained using Nomis (https://www.nomisweb.co.uk/).

The univariate relationship between genomic cluster status and each of the characteristics listed above was modelled separately using binary logistic regression. Results are reported as odds ratios (OR) with 95% confidence intervals. A multivariable binary logistic regression was also used to model the relationship between genomic cluster membership and each potential factor associated with genomic clustering, whilst adjusting for the remaining potential factors. Results are reported as adjusted odds ratios (aOR) with 95% confidence intervals.

We conducted a further analysis to model the relationship between cases’ treatment outcomes (favourable [completed treatment] versus unfavourable [died, lost to follow-up, treatment stopped]) and their genomic clustering status (clustered or non-clustered) whilst accounting for the other case characteristics listed above. Logistic regression analyses conducted using small-to-medium samples can result in infinite parameter estimates if responses/non-responses can be perfectly separated by individual factors. In such circumstances, a maximum likelihood parameter estimate does not exist. In this further analysis, the response was indeed perfectly separated by individual factors. We therefore used multivariable Firth’s regression, which uses penalised maximum likelihood estimation to generate finite parameter estimates and is suitable for use in the context of separation^28^. We used multivariable Firth’s regression, via the logistf package^29^. Results are reported as adjusted odds ratios with 95% confidence intervals.

Data analysis and mapping were undertaken in R Studio (https://posit.co/download/rstudio-desktop/; R V4.1.3) and PowerBI (https://powerbi.microsoft.com/en-us/blog/power-bi-june-2025-feature-summary/; v2.144.1155.0 64-bit (June 2025)), respectively. P values of < 0.05 were considered statistically significant.

## Results

1,141 notified TB cases met the inclusion criteria across the study period. Of these, 1,096 (96%) were Welsh individuals treated in Wales and thus information on culture positivity was available. Samples from 796 of those were culture positive (72.6%), with 280 of those (35.2%) subsequently being sequenced. Of all cases that were not sequenced (*n* = 1,141–280 = 861), 689 were culture positive (80%).

This sequencing coverage may, however, be an underestimation due to data linkage constraints. In addition, WGS for TB did not become routine in Wales until 2019, so prior to this point, only a subset of samples were sequenced, primarily for testing and validation purposes. From 2019 onwards, the PenGU routinely sequenced culture positive samples whenever possible, such that sequencing coverage has subsequently improved. Indeed, of the 1,096 patients treated in Wales over the study period, 317 (28.9%) were notified from 2019 onwards, and samples from 250 of those were culture positive (78.9%), of which 203 were subsequently sequenced (81.2%). Of the 114 cases since 2019 that were not sequenced, 90 were culture-positive (78.9%).

**Fig. 1:**
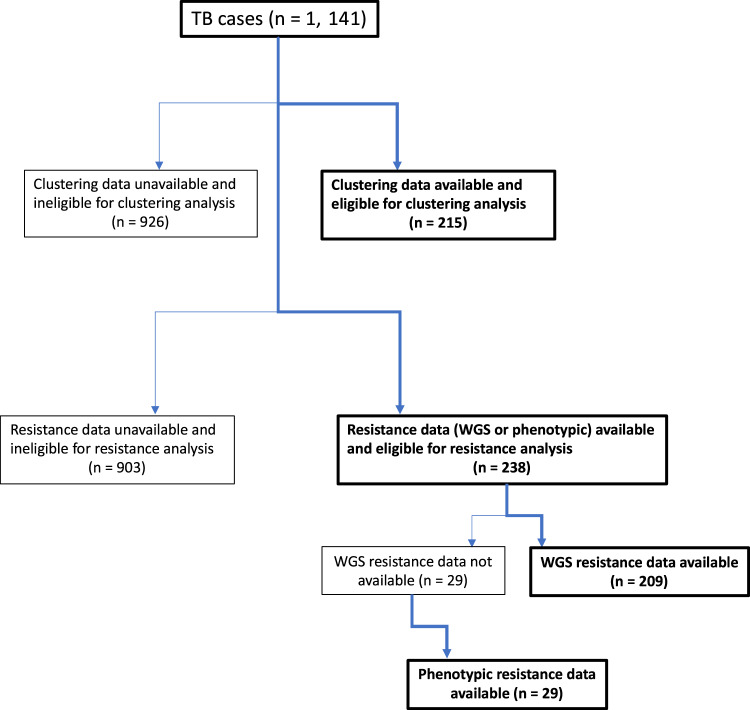
Flowchart of cases eligible and included in the clustering analysis and cases eligible and included in the resistance analysis of all eligible TB cases over the study period.

Different subsets of the sequenced cases had subsequently been submitted to the UKHSA drug resistance and clustering pipelines. Consequently, of the 1,141 total notified TB cases, 215 (18.8%) were eligible for the clustering analysis reported below, and 238 (20.9%) were eligible for the resistance profile analysis (Fig. 1).

### Sample demographics/characteristics

**Fig. 2:**
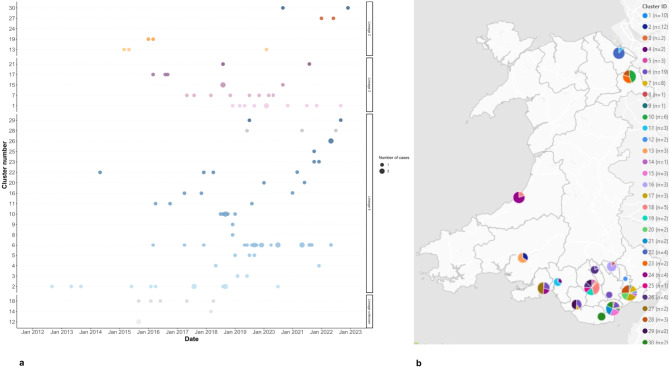
(**a**) Timeline of genotypic TB clusters in Wales, 2012–2022. Clusters are ordered on the y-axis by TB lineage, and the number of cases is indicated by the size of the dots. TB Lineages are given by the point colours: Lineage 2 = orange; Lineage 3 = purple; Lineage 4 = blue; Lineage unknown = grey. (**b**) Map showing the number of genotypic clusters per local authority, in Wales, 2012–2022. Pie chart size indicates the number of cases, colour indicates the cluster ID. Note: A small number of clusters are shown as comprising *N* = 1 cases over the study period because other linked cases were notified outside the study window or could not be retrieved from NTBS.

115/215 included cases were part of 30 genomic clusters. These clusters belonged to Lineage 2 (*n* = 5), Lineage 3 (*n* = 5), and Lineage 4 (*n* = 17) (Fig. 2a). The lineage was unknown for three clusters. Case dates ranged from 2012 to 2022 with the majority of cases from 2019 onwards. Clusters belonging to Lineage 2 were the smallest in size, with a maximum of four cases per cluster. The maximum cluster size in Lineage 3 was 10 cases, whilst for Lineage 4 it was 19. Most clusters/cases were located in local authorities in South Wales, with the exception of five clusters in North Wales, and four in West Wales (Fig. 2b).

**Fig. 3:**
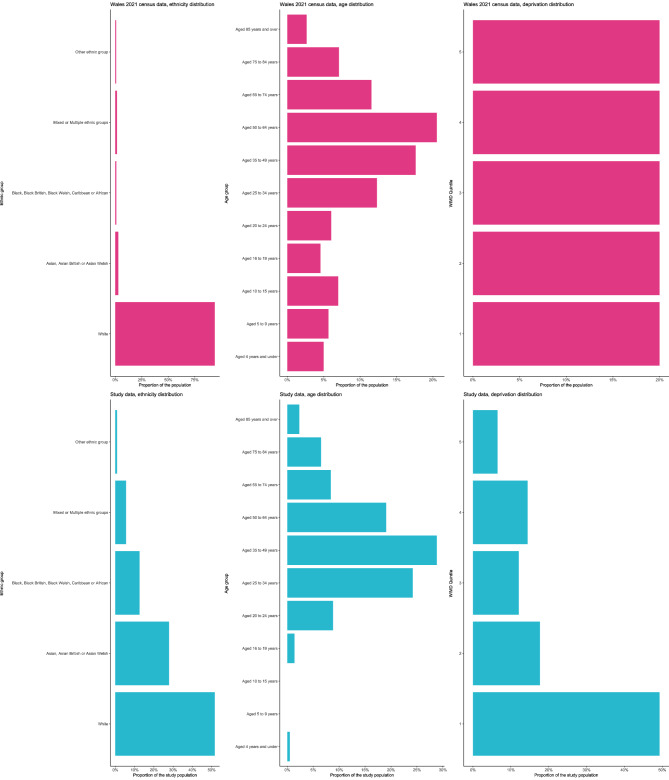
Distribution of ethnic groups, age groups and WIMD in the study population (blue) vs. the Welsh population (Census data from 2021) (pink). Note: Unknowns within the study data have not been visualised, counts can be found within Table 1.

The characteristics of the study population and the sub-population that were part of a genomic cluster are summarised in Table 1. Approximately two thirds of the cases, and cases part of a genomic cluster, were male. The largest proportion of cases, approximately a quarter, were aged 25–34. The same was true of those in a genomic cluster although a similar number were aged 35–44 and 45–54. Few cases were under 19 years of age. There was a significant difference between the sample and Welsh population distributions for age group (X^*2*^ = 466.02, *p* < 0.001, Fig. 3).

**Table 1:** Demographic and clinical characteristics of genomic clustered and non-clustered cases in wales, united Kingdom 2012–2022. Univariate and multivariable regression analysis on the factors associated with genomic clustering.

Characteristic		Total cases n (%)	In a genomic cluster n (%)	Not in a genomic cluster n (%)	Cluster size range	Fishers exact (p)	Univariate regression		Multivariable regression	
							OR^1^ (95% CI^2^)	p	aOR^3^ (95% CI)	p
Sex	Female	73 (34%)	37 (32.2%)	36 (36%)	2 to 19	0.567	.	.	.	.
	Male	142 (66%)	78 (67.8%)	64 (64%)	1 to 19		1.19 (0.67–2.09)	0.6	1.58 (0.65–3.94)	0.3
Age group	0–18	3 (1.4%)	2 (1.7%)	1 (1%)	2 to 6	0.104	2.16 (0.2–48.2)	0.5	6.63 (0.27–376)	0.3
	19–24	20 (9.3%)	12 (10.4%)	8 (8%)	1 to 19		1.62 (0.57–4.76)	0.4	0.5 (0.12–2.07)	0.3
	25–34	52 (24.2%)	25 (21.7%)	27 (27%)	2 to 19		.	.	.	.
	35–44	38 (17.7%)	25 (21.7%)	13 (13%)	1 to 19		2.08 (0.89–5.02)	0.1	2.52 (0.66–10.4)	0.2
	45–54	38 (17.7%)	25 (21.7%)	13 (13%)	1 to 19		2.08 (0.89–5.02)	0.1	1.13 (0.31–4.12)	0.8
	55–64	27 (12.6%)	12 (10.4%)	15 (15%)	2 to 12		0.86 (0.34–2.2)	0.8	0.38 (0.09–1.49)	0.2
	65+	37 (17.2%)	14 (12.2%)	23 (23%)	2 to 12		0.66 (0.27–1.54)	0.3	0.3 (0.08–1.09)	0.072
Country of Birth	Born outside of the UK	99 (46%)	30 (26.1%)	69 (69%)	1 to 19	**< 0.001**	.	.	.	.
	Born in UK	108 (50.2%)	78 (67.8%)	30 (30%)	1 to 19		5.98 (3.32–11.1)	**< 0.001**	14.1 (4.67–47.8)	**< 0.001**
	Unknown	8 (3.7%)	7 (6.1%)	1 (1%)	2 to 19		16.1 (2.7–308)	**0.011**	28.2 (2.64–831)	**0.016**
Ethnic group	White	111 (51.6%)	70 (60.9%)	41 (41%)	1 to 19	**0.008**	.	.	.	.
	Asian–Other	5 (2.3%)	3 (2.6%)	2 (2%)	2 to 4		0.88 (0.14–6.88)	0.9	3.37 (0.25–67.7)	0.4
	Bangladeshi	5 (2.3%)	1 (0.9%)	4 (4%)	2 to 2		0.15 (0.01–1.03)	0.091	0.22 (0-7.58)	0.4
	Black	27 (12.6%)	11 (9.6%)	16 (16%)	1 to 19		0.4 (0.17–0.94)	**0.038**	2.21 (0.49–10.5)	0.3
	Chinese	5 (2.3%)	3 (2.6%)	2 (2%)	4 to 4		0.88 (0.14–6.88)	0.9	5.83 (0.6–65.7)	0.13
	Indian	12 (5.6%)	1 (0.9%)	11 (11%)	19 to 19		0.05 (0-0.29)	**0.006**	0.64 (0.02–6.83)	0.7
	Mixed/Other	14 (6.5%)	6 (5.2%)	8 (8%)	1 to 19		0.44 (0.14–1.35)	0.2	1.94 (0.32-12)	0.5
	Pakistani	33 (15.3%)	18 (15.7%)	15 (15%)	2 to 10		0.7 (0.32–1.56)	0.4	1.62 (0.44–6.31)	0.5
	Unknown	3 (1.4%)	2 (1.7%)	1 (1%)	2 to 19		1.17 (0.11–25.7)	0.9	6.84 (0.28–345)	0.3
WIMD 2019 overall quintile	1	106 (49.3%)	60 (52.2%)	46 (46%)	1 to 19	**0.027**	.	.	.	.
	2	38 (17.7%)	21 (18.3%)	17 (17%)	2 to 19		0.95 (0.45–2.01)	0.9	0.83 (0.29–2.34)	0.7
	3	26 (12.1%)	7 (6.1%)	19 (19%)	2 to 19		0.28 (0.1–0.7)	**0.009**	0.24 (0.6–0.86)	**0.032**
	4	31 (14.4%)	21 (18.3%)	10 (10%)	1 to 19		1.61 (0.7–3.88)	0.3	2.51 (0.79–8.58)	0.13
	5	14 (6.5%)	6 (5.2%)	8 (8%)	2 to 8		0.58 (0.18–1.77)	0.3	0.56 (0.11–2.77)	0.5
Social factors potentially associated with genomic clustering	No	129 (60%)	58 (50.4%)	71 (71%)	1 to 19	**0.002**	.	.	.	.
	Yes	66 (30.7%)	47 (40.9%)	19 (19%)	1 to 19		3.03 (1.62–5.82)	**< 0.001**	0.88 (0.32–2.33)	0.8
	Unknown	20 (9.3%)	10 (8.7%)	10 (10%)	2 to 19		1.22 (0.47–3.18)	0.7	0.95 (0.22–4.02)	> 0.9
HIV	No	186 (86.5%)	102 (88.7%)	84 (84%)	1 to 19	0.618	.	.	.	.
	Yes	4 (1.9%)	2 (1.7%)	2 (2%)	1 to 2		0.82 (0.1–6.98)	0.8	0.35 (0.02–6.22)	0.5
	Unknown	25 (11.6%)	11 (9.6%)	14 (14%)	2 to 19		0.65 (0.27–1.5)	0.3	0.27 (0.09–0.83)	**0.023**
Diabetes	No	187 (87%)	105 (91.3%)	82 (82%)	1 to 19	0.066	.	.	.	.
	Yes	28 (13%)	10 (8.7%)	18 (18%)	5 to 12		0.43 (0.18–0.97)	**0.047**	0.52 (0.13–2.02)	0.3
Hepatitis B	No	211 (98.1%)	114 (99.1%)	97 (97%)	1 to 19	0.34	.	.	.	.
	Yes	4 (1.9%)	1 (0.9%)	3 (3%)	8 to 8		0.28 (0.01–2.26)	0.3	0.07 (0-1.32)	0.1
Hepatitis C	No	208 (96.7%)	109 (94.8%)	99 (99%)	1 to 19	0.125	.	.	.	.
	Yes	7 (3.3%)	6 (5.2%)	1 (1%)	3 to 19		5.45 (0.91–104)	0.12	7.44 (0.43–359)	0.2
Immunosuppression	No	199 (92.6%)	108 (93.9%)	91 (91%)	1 to 19	0.446	.	.	.	.
	Yes	16 (7.4%)	7 (6.1%)	9 (9%)	1 to 12		0.66 (0.23–1.83)	0.4	0.71 (0.14–3.42)	0.7
Liver disease	No	210 (97.7%)	113 (98.3%)	97 (97%)	1 to 19	0.665	.	.	.	.
	Yes	5 (2.3%)	2 (1.7%)	3 (3%)	6 to 6		0.57 (0.07–3.52)	0.5	0.35 (0.02–4.47)	0.4
Kidney disease	No	209 (97.2%)	113 (98.3%)	96 (96%)	1 to 19	0.420	.	.	.	.
	Yes	6 (2.8%)	2 (1.7%)	4 (4%)	2 to 10		0.42 (0.06–2.23)	0.3	0.47 (0.03–5.64)	0.5
Site of Disease	Extra pulmonary	24 (11.2%)	6 (5.2%)	18 (18%)	1 to 19	**< 0.001**	.	.	.	.
	Pulmonary	175 (81.4%)	105 (91.3%)	70 (70%)	1 to 19		4.5 (1.79–12.9)	**0.002**	6.81 (1.72–29.9)	**0.008**
	Pulmonary + other sites	16 (7.4%)	4 (3.5%)	12 (12%)	3 to 19		1 (0.22–4.28)	> 0.9	0.51 (0.06–3.7)	0.5
Previous infection	No	185 (86%)	99 (86.1%)	86 (86%)	1 to 19	0.550	.	.	.	.
	Yes	14 (6.5%)	9 (7.8%)	5 (5%)	2 to 19		1.56 (0.52–5.25)	0.4	1.34 (0.21–9.26)	0.8
	Unknown	16 (7.4%)	7 (6.1%)	9 (9%)	1 to 8		0.68 (0.23–1.89)	0.5	1.61 (0.28–8.79)	0.6

^*1*^*OR = Odds Ratio*, ^*2*^*CI = Confidence Interval*, ^*3*^*aOR = adjusted Odds Ratio*.

Over two thirds of cases part of a genomic cluster were UK-born despite overall, a similar number of cases being born outside of the UK. Over half of cases, and cases part of a genomic cluster, were White. There was a significant difference between the sample and Welsh population distributions for ethnicity (X^2^ = 7635.9, *p* < 0.001, Fig. 3), reflecting the disproportionate representation of non-White ethnic groups in our study sample.

Almost half of the cases, and more than half of the cases that were part of a genomic cluster lived within WIMD quintile 1 (the most deprived). The fewest number of cases lived within in WIMD quintile 5 (least deprived). There was a significant difference between the sample and Welsh population distributions for WIMD quintile (X^*2*^ = 122.51, *p* < 0.001, Fig. 3), reflecting the disproportionate representation of individuals living within WIMD quintile 1.

Over a quarter of cases had at least one social factor potentially associated with genomic clustering (imprisonment, homelessness status, drug misuse, and/or alcohol misuse); more than two thirds of these cases belonged to a genomic cluster. More than 40% of all cases which were part of a genomic cluster had any social factor potentially associated with genomic clustering. Most cases, and cases part of a genomic cluster, had pulmonary TB.

### Factors associated with genomic clustering

Fisher’s exact tests (Table 1) revealed that for country of birth, ethnic group, WIMD quintile, any social factor potentially associated with genomic clustering, diabetes, and site of disease, there was a statistically significant relationship with being part of a genomic cluster (Table 1).

In the univariate analysis, the odds of being in a genomic cluster were greater for cases with any social factor potentially associated with genomic clustering, compared to those without (OR = 2.98, 95% CI = [1.6–5.71], *p* < 0.001). The odds of being in a genomic cluster were lower for cases who were Indian, or Black, compared to those who were white, and cases with diabetes, compared to those without (OR = 0.05, 95% CI = [0–0.29], *p* = 0.006); OR = 0.4, 95% CI = [0.17–0.94], *p* = 0.038; OR = 0.43, 95% CI = [0.18–0.97], *p* = 0.047).

The odds of being in a genomic cluster for cases born in the UK were increased in both the univariate and multivariate model, compared to those born outside of the UK, (aOR = 14.1, 95% CI = [4.67–47.8], *p* < 0.001). This was also true for cases with pulmonary TB, compared to those with extra-pulmonary TB (aOR = 6.81, 95% CI = [1.72–29.9], *p* = 0.008). The odds of being in a genomic cluster for cases living in WIMD quintile 3 were decreased in both the univariate and multivariate model, compared to those living in quintile 1, (aOR = 0.24, 95% CI = [0.06–0.86], *p* = 0.032).

Finally, 208 cases were included in the treatment outcome analysis; seven cases were excluded as outcome was unknown. The aOR of having a favourable outcome when part of a genomic cluster was not significantly different. A statistically significant result was observed, however, for those who were immunosuppressed (aOR = 0.16 95% CI = [0.03–0.61], *p* = 0.007), and whose HIV status was unknown.

### Drug resistance analysis

**Fig. 4:**
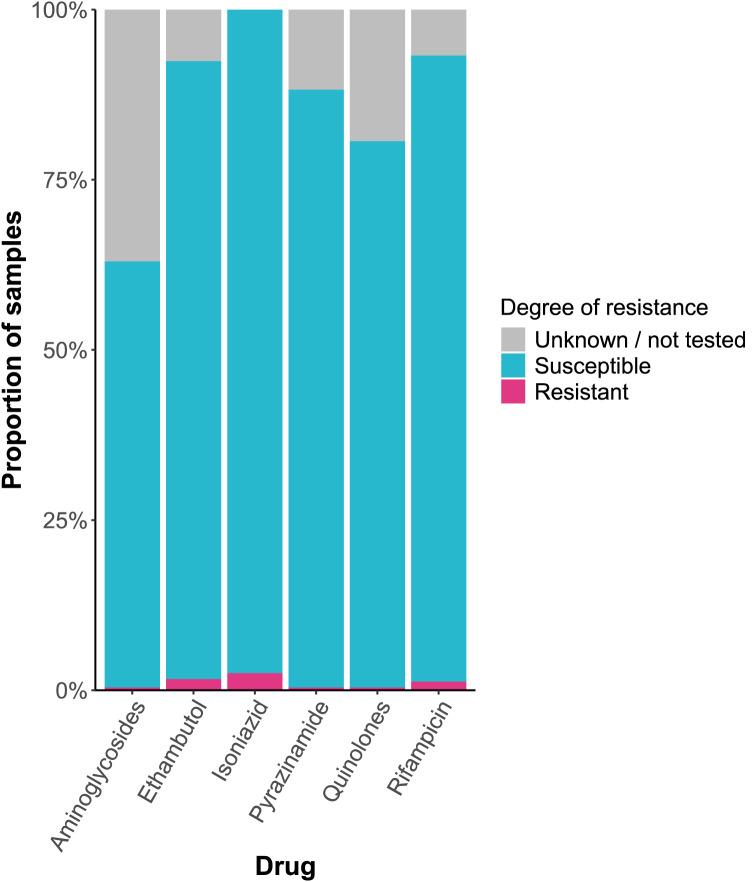
Resistance profile of Mycobacterium tuberculosis in Wales, 2014–2022.

Resistance/predicted resistance was low amongst all drugs: isoniazid (2.52%), ethambutol (1.68%), rifampicin (1.26%), aminoglycosides (0.42%), pyrazinamide (0.42%), and quinolone (0.42%). The proportion of cases for which the degree of resistance or predicted resistance was unknown or not tested ranged from 0 to 36.97% (Fig. 4). One case was MDR, but there were no cases of pre-XDR or XDR TB.

## Discussion

This retrospective study of the distribution and genomic epidemiology of Welsh TB cases revealed that most clustered cases were Lineage 4 and only a small proportion of sequenced/typed cases demonstrated predicted drug-resistance. Clustered cases were mainly concentrated in the densely populated region of South Wales, and we identified significant differences in the distribution of ethnicity, age group, and WIMD deprivation quintile in our sample compared to the general population of Wales. To our knowledge, this is the first study investigating factors associated with TB genomic clustering in Wales. Factors that were associated with increased odds of genomic cluster membership included being UK-born and pulmonary disease.

Most clusters belonging to Lineage 4 suggests the distribution of TB lineages within Wales has remained similar to the UK-wide distribution described by^7^ between 2010 and 2015 and^8^ between 2009 and 2019. It is reassuring that a smaller proportion of cases belong to Lineage 2, which is strongly associated with drug resistance strains, including MDR and XDR^30^. Whilst predicted resistance was very low for each drug, the predicted resistance status was unknown (i.e., the software for WGS-based genotypic testing could not determine the resistance level, or the resistance pipeline quality control criteria were not met *and* no phenotypic testing data was available) or not tested for up to over a third of cases, casting uncertainty on the extent of TB drug resistance within Wales. However, the findings of a single predicted MDR case and a rifampicin resistance rate of 1.26%, a lower prevalence than other parts of the UK, and low rate of predicted resistance to quinolones and aminoglycosides, second-line TB drugs, are particularly encouraging^2,16^.

Our finding of an increased odds of genomic cluster membership for those whose disease was pulmonary, compared to extra-pulmonary, supports previous findings^7–9^. People with extra-pulmonary disease are rarely infectious and therefore our finding may be attributable to the increased transmissibility of the pulmonary disease form^31,32^. Individuals who were immunosuppressed had a decreased odds of having a favourable treatment outcome possibly due to diagnosis difficulties in immunocompromised patients owing to their differing clinical presentation and atypical symptoms^33,34^. We suggest immunocompromised individuals suspected of belonging to a TB genomic cluster are prioritised for diagnosis to prevent delayed treatment and improve patient outcomes.

The pattern of over- and under-representation of particular age, ethnic, and WIMD quintile groups in our study sample relative to the general population of Wales (see Fig. 3), highlights important health inequalities in the burden of TB. The pattern observed here is broadly consistent with the findings of^35^ who report the number of TB notifications in England was highest in individuals aged 25–34 and lowest in children aged < 18 years. They also reported that while absolute notification numbers were highest in individuals recorded as belonging to White ethnic groups, the highest notification rates were among non-White ethnic groups, and notification rates were also found to increase with increasing levels of deprivation based on the IMD. The majority of both clusters and larger clusters being distributed within South Wales is consistent with the increased population density within these cities and increased social mixing and opportunity for transmission. Indeed, compared to the Health Boards comprising Mid, North and West Wales (Betsi Cadwaladr, Hywel Dda and Powys Teaching Health Board), the Health Boards comprising South Wales (Cardiff and the Vale, Aneurin Bevan, Cwm Taf Morgannwg, and Swansea Bay) generally have a higher TB incidence (0.7, 1.6 and 3.0 versus 4.4, 4.4, 0.2 and 3.7 cases per 100k population, respectively). Individuals living in more deprived (as defined by the IMD in previous studies) and/or heavily populated areas may also live in overcrowded, possibly poorly ventilated dwellings and have a poorer nutritional status, both of which are associated with higher risk of TB infection as well as development to TB disease^36,37^.

Our multivariate logistic regression analysis also revealed a decreased odds of being in a genomic cluster for those living in WIMD quintile 3, compared to WIMD quintile 1 (the most deprived quintile). A corresponding significantly reduced odds of cluster membership might also be expected in WIMD quintiles 4 and 5 (the least deprived quintiles), though this was not borne out in our data. The absence of such effects here should, however, be interpreted with caution, especially in the context of our modest sample size, because WIMD deprivation quintiles are assigned to Welsh LSOAs rather than individuals. It is therefore possible that despite living in an LSOA characterised by relatively low deprivation, some of the cases in our WIMD quintile 4 and 5 groups may personally experience significant deprivation.

We found that those that were UK-born were more likely to be part of a genomic cluster, which is in agreement with numerous studies investigating the prevalence of TB in low incidence countries^7–9,11,21^. Many patients born outside the UK may not be involved in local transmission clusters but rather may reactivate (latent) infections acquired in their birth country^8,21^. Genomic cluster links to non-UK patients would not, however, be identifiable in our national dataset. Alternatively, this finding may be due to later diagnoses, and subsequent transmission, among those born in UK compared to those born outside of the UK who may receive a diagnosis prior to becoming symptomatic due to undergoing pre-entry screening and TB infection screening^7,8^. Viewed from the opposite perspective, those born outside of the UK may be subject to greater clinical suspicion of TB and the resulting faster diagnosis may lead to reduced transmission and smaller clusters. Another possible explanation may be related to our finding that of those in a genomic cluster, 44% of UK-born individuals had a social factor (e.g., homelessness, imprisonment, alcohol and/or substance misuse) compared with 17% of individuals born outside of the UK. Such social factors may result in inadequate health-care-seeking behaviours causing greater periods of infectivity and the potential for onward transmission^21^.

Current preventative strategies for TB in Wales and the UK generally focus on new entrant screening, despite UK national guidance recommending systematic screening for TB infection in underserved populations^38^. Indeed, a potential need to augment existing preventative measures with targeted screening and controlling of TB within disadvantaged groups, could warrant further analysis. Such targeted screening of onset cases (in a genomic cluster) within these groups may potentially enable early detection and minimise onward spread. Research is, however, needed to assess the suitability, feasibility, and cost-effectiveness of such additional preventative measures, as this was beyond the scope of the present work.

### Limitations

A limitation was the small sample size with incomplete sequencing coverage of TB cases sampled over a relatively short recent time period in Wales, which may have reduced representativeness and statistical power. In addition, though we endeavoured to include sequencing/clustering data linked to all samples available for sequencing across the study period in our analyses, sequencing coverage varied over the study period. This is because samples have been routinely sequenced by the PenGU, whenever possible, since the routine TB sequencing service was established in 2019, reflected in the high sequencing coverage of > 80% we report in the period since 2019. This is substantially higher than the sequencing coverage observed over the whole study period because retrospective sequencing of the pre-2019 samples was contingent on availability of a viable frozen sample in storage, again potentially affecting our sample/cluster representativeness etc. It is also important to highlight that it was not possible to retrospectively retrieve data for all Welsh cases involved in potential transmission clusters identified by the UKHSA FindNeighbours pipeline, if they had not been notified and recorded correctly in NTBS. This data capture/linkage limitation accounts for the small number of clusters depicted as comprising a single case in Fig. 2, other than cluster 25, which involved additional cases that were notified prior to our study window. This data linkage issue may, however, also have affected the estimated size of the larger clusters.

The proximity of many of the high TB incidence local authorities to the English border, where daily migration between the countries for work occurs, increases the potential of clustering with English cases. No contextual samples from England were available for analysis and cross-border clustering patterns were beyond the scope of the present analysis. We aim to address both these limitations in future, in part, by collaborating further with the four UK nations to update the TB clustering service and data sharing practices. This will enable us to augment the existing dataset with UK-wide contextual samples and provide a broader understanding of the distribution, transmission and genomic clustering of Welsh TB cases. Additionally, this will enhance our existing routine TB surveillance and outbreak investigation capabilities.

Another limitation of the present study was the lack of additional retrospective epidemiological linkage data. Future work undertaking this further data linkage could corroborate the genomic clustering results, but also investigate the epidemiological links between the putative transmission events that they suggest, as per^21^.

Data availability within our study impacted our ability to draw inferences about the extent of TB drug resistance within Wales; for some drugs, resistance could not be predicted in more than a third of cases due to the high level of data quality required for WGS-based resistance prediction. Missing data also impacted our genomic clustering analysis, including the finding that the odds of membership of a genomically-confirmed cluster are significantly reduced in individuals with an unknown HIV status, which contrasted to that of^39^. Such findings are difficult to interpret and suggest scope for bias in our parameter estimates. Nevertheless, recognising the ubiquity of missing data in epidemiological research, further refinements of our analyses could include using multiple imputation to mitigate bias and information loss arising from missing data.

## Conclusion

Our study revealed important health inequalities in the burden of TB in Wales and that socio-economic deprivation continues to play a substantial role in sustaining the spread of TB in Wales, especially in the UK-born population. This supports reiteration of existing UK national guidance recommending systematic screening for TB infection in underserved populations. Further research could consider development of additional screening/control measures when the onset case in a genomic cluster has one or more of these characteristics, which could potentially improve diagnosis and access to primary healthcare, preventing further spread and improving patient outcomes.

## Data Availability

Aggregate data available on request due to privacy/ethical restrictions. Requests for data should be directed to the corresponding author via PHW.GenomicEpi@wales.nhs.uk.
